# GRIEF FOLLOWING A COVID-19 DEATH

**DOI:** 10.1093/geroni/igad104.2665

**Published:** 2023-12-21

**Authors:** Karuna Thomas, Nancy Pachana, Hannah Silverstein, Chayna Kanofsky, Hyun Jin Cho, Brian Carpenter

**Affiliations:** Washington University in St. Louis, St. Louis, Missouri, United States; The University of Queensland, Brisbane, Queensland, Australia; Washington University in St. Louis, St. Louis, Missouri, United States; Washington University in St. Louis, St. Louis, Missouri, United States; Washington University in St Louis, St. Louis, Missouri, United States; Washington University in St. Louis, St. Louis, Missouri, United States

## Abstract

The large number of deaths due to COVID-19 has resulted in a similarly high number of people experiencing grief. Attending to the needs of grieving individuals will be a critical public health issue for years to come. Moreover, the circumstances surrounding many COVID-19 deaths may contribute to more severe and protracted grief. The aim of this qualitative study was to learn about the social and psychological experiences of pandemic-related grief. Semi-structured interviews were conducted with individuals who had lost someone close to them to COVID-19 (N = 25). Interview questions addressed circumstances surrounding the death, rituals and memorializations undertaken, and subsequent emotional consequences. Thematic analysis was used to identify themes and patterns. Participants described their grief as being highly personal and unique and only understood by others who had experienced a similar loss from COVID-19. They expressed significant distress at the circumstances surrounding the death, with many not having an opportunity to say goodbye. Their distress was amplified by a lack of closure due to limited rituals allowed during the pandemic. Participants also reported feeling invisible in their experience of loss and grief as they reflected on the overwhelmed healthcare systems during the height of the pandemic, comparison to the type of experience they would have had were it not for the pandemic, as well as feeling the world had moved on once pandemic restrictions were lifted. Taken together, these findings suggest unique grief experiences with COVID-19 and the need for targeted interventions from mental health practitioners.

